# Medicinal Chemistry of Drugs with *N*-Oxide Functionalities

**DOI:** 10.1021/acs.jmedchem.4c00254

**Published:** 2024-03-29

**Authors:** Michelle Kobus, Timo Friedrich, Eilika Zorn, Nils Burmeister, Wolfgang Maison

**Affiliations:** Universität Hamburg, Department of Chemistry, Bundesstrasse 45, 20146 Hamburg, Germany

## Abstract

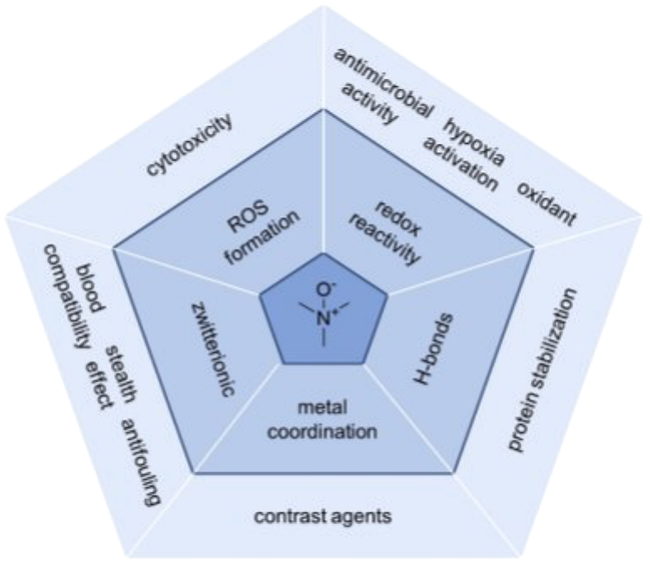

Molecules with *N*-oxide functionalities are omnipresent in nature and play an important role in Medicinal Chemistry. They are synthetic or biosynthetic intermediates, prodrugs, drugs, or polymers for applications in drug development and surface engineering. Typically, the *N*-oxide group is critical for biomedical applications of these molecules. It may provide water solubility or decrease membrane permeability or immunogenicity. In other cases, the *N*-oxide has a special redox reactivity which is important for drug targeting and/or cytotoxicity. Many of the underlying mechanisms have only recently been discovered, and the number of applications of *N*-oxides in the healthcare field is rapidly growing. This Perspective article gives a short summary of the properties of *N*-oxides and their synthesis. It also provides a discussion of current applications of *N*-oxides in the biomedical field and explains the basic molecular mechanisms responsible for their biological activity.

## Significance

Relevance of *N*-oxides for Medicinal Chemistry:

- N^+^–O^–^ bonds are highly polar and form strong hydrogen bonds. They may be inert or reactive in biological systems depending on their substituents.
- *N*-Oxide groups can be used to increase the solubility of drugs and decrease membrane permeability.
- Many heteroaromatic and aniline-derived *N*-oxides are reduced enzymatically in vivo and find applications as hypoxia-activated prodrugs.
- Polymeric *N*-oxides have excellent blood compatibility, are nonimmunogenic and are nonadhesive for microorganisms (stealth materials).

## 1 Introduction

**Figure 1:**
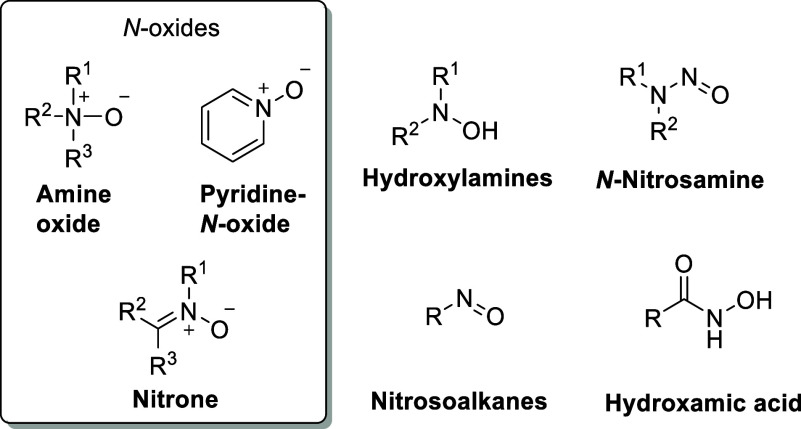
*N*-Oxides and other oxygenated organic nitrogen species.

Oxides of tertiary amines (amine oxides, Figure 1) and aromatic amines (e.g., pyridine-*N*-oxide, Figure 1) are typically summarized as *N*-oxides. They should not be confused with other oxygenated nitrogen species, such as hydroxylamines or nitroso compounds (Figure 1). Oxides of imines (nitrones, Figure 1) can also be considered *N*-oxides but have other characteristic chemical properties compared to amine- and pyridine-*N*-oxides.

Molecules with *N*-oxide functionalities are omnipresent in nature. An example is trimethylamine-*N*-oxide (TMAO),^1,2^ a compound of possible relevance for cancer and cardiovascular diseases in humans and a protein stabilizer in seawater fish. In addition, many *N*-oxides are nontoxic derivatives of corresponding amines and are thus common metabolites of drugs and natural products.^3,4^ Synthetic *N*-oxides are increasingly used in various healthcare-related applications.^5^ Recent examples include reagents for magnetic resonance imaging,^6^ prodrugs,^7^ targeted cytotoxic^8^ and antibacterial drugs.^9^ Furthermore, oligomeric *N*-oxides have the potential to serve as substitutes for other hydrophilic oligomers and polymers, such as polyethylene glycol (PEG). Recent studies revealed that these oligomeric *N*-oxides have excellent blood compatibility^10^ and can be used as stealth reagents for the conjugation to drugs or material surfaces.^11^ As many of the underlying principles have only recently been discovered,^12^ the number of applications of *N*-oxides in the healthcare field is currently rapidly growing. Many applications rely on the stealth character of *N*-oxides, requiring chemical inertness in biological systems.^13^ Others make use of their redox reactivity,^14,15^ while the overlap of both opens additional fields of applications.^16^ Both redox activity and stealth character depend on the unique N^+^–O^–^ bond and the resulting zwitterionic character of *N*-oxides.^17^ However, a molecular understanding of *N*-oxides is limited among many researchers in the biomedical field. This knowledge is essential to designing drugs with a tailored biological activity and therefore a central teaching aspect of this Perspective.

The article covers an introduction to the chemical and physical properties of the *N*-oxide functionality. The synthesis of *N*-oxides and physiologically relevant reactions of *N*-oxides are also highlighted. A summary of applications in Medicinal Chemistry will be given and a critical discussion provided.

## 2 Properties of *N*-Oxides

**Figure 2:**
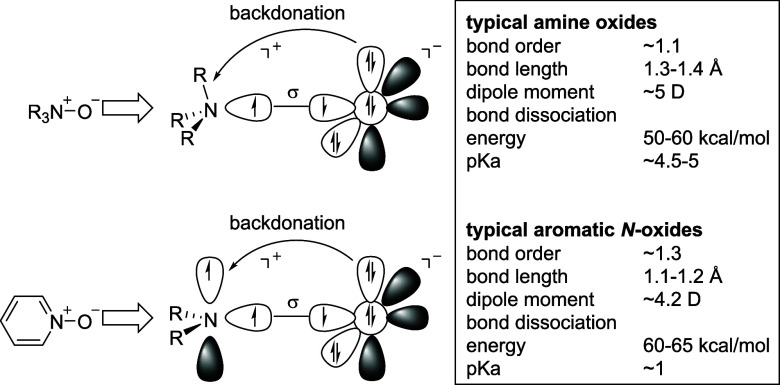
Comparison of the N^+^–O^–^ bond characteristics and selected parameters for amine oxides (R_3_NO) and aromatic *N*-oxides (with pyridine-*N*-oxide as an example).

*N*-Oxides contain a dative and highly polar N^+^–O^–^ bond with a bond order significantly higher than one. It is best described by a single N^+^–O^–^ bond with important contributions of O → N backdonation (Figure 2). The latter is dependent on the nature of the substituents attached to nitrogen and its hybridization. In amine oxides (R_3_NO), the N^+^–O^–^ bond order is only slightly higher than one (e.g., 1.1 for TMAO)^18^ with a low but significant hyperconjugative contribution of LP_O_ → σ*_NR_. Higher bond orders of 1.3 are typical for aromatic *N*-oxides with a stronger π-backdonation of LP_O_ → π*_CN_.^19^ Aromatic *N*-oxides are thus characterized by a slightly shorter and more stable N^+^–O^–^ bond.^20^ This is reflected by the reactivity trend: amine oxides are more easily reduced than aromatic *N*-oxides.^21^ The dipole moment of the N^+^–O^–^ bond is large with values of 4.0 to 5.0 D. Amine oxides are typically more polar than aromatic *N*-oxides (e.g., 5.02 D for TMAO vs 4.28 D for pyridine-*N*-oxide).^22^ These values are significantly larger than those of other polar bonds, e.g., P–O, P–S, or S–O.^23^

*N*-Oxides are weak bases and may be protonated to stable hydroxyammonium species with typical p*K*_a_ values of 4–5 for amine oxides^21^ and 0.5–2 for aromatic *N*-oxides.^24^ The zwitterionic net neutral form is thus dominant at physiological pH. *N*-Oxides are stabilized by polar protic solvents and are often isolated as hydrates, due to their hygroscopic character.^25^ They form remarkably stable hydrogen bonds with water and alcohols, which is the reason for a number of special properties: *N*-methyl-morpholine-*N*-oxide (NMO), for example, can dissolve cellulose and is thus used in the manufacturing of cellulose fibers in the Lyocell process.^26^ NMO and other *N*-oxides are important oxidants in synthetic organic chemistry, for example in Upjohn or Ley–Griffith oxidations.^27,28^ Interestingly, their tendency to form stable hydrogen bonds has also been used in this context.^29^ An example is the stabilization of carbonyl-hydrates by NMO, which is an important feature of a TPAP-catalyzed direct oxidation protocol of alcohols to carboxylic acids.^30^

TMAO is a naturally occurring osmolyte known to stabilize proteins at neutral pH. It counteracts the denaturing effects of salts, urea, and hydrodynamic pressure in a pH range from 6 to 8, a feature used in nature and in biotechnology.^31^ At pH values lower than 5, TMAO is protonated to a large extent and destabilizes protein structure.^32^

**Figure 3:**
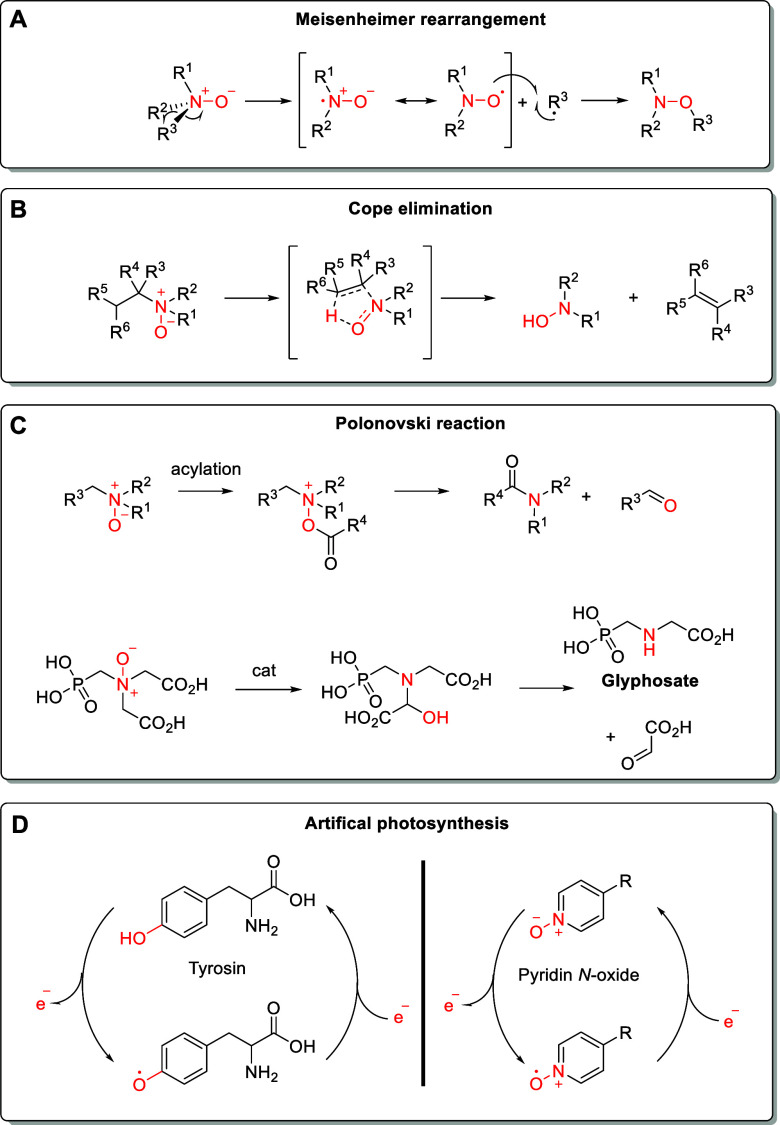
Reactivity of *N*-oxides: A) Meisenheimer rearrangement; B) Cope elimination; C) Polonovski reaction; and D) pyridine-*N*-oxides as electron shuttles.

*N*-Oxides are generally stable at room temperature. However, amine oxides may be prone to decomposition and scaffold rearrangements at higher temperatures (above ∼100 °C for TMAO, ∼150 °C for aromatic *N*-oxides),^33−35^ in the presence of electrophiles or transition metals.^36^ Typical reactions involved are Meisenheimer rearrangements (particularly for *N*-allyl and *N*-benzyl derivatives, Figure 3A),^37^ Cope eliminations (Figure 3B),^38^ or Polonovski reactions (Figure 3C).^39^ The latter are *syn*-eliminations and require the presence of a hydrogen atom in the β-position to the *N*-oxide group. Polonovski reactions are classically triggered by *N*-*O*-acylation with reactive acyl compounds such as acyl halogenides, anhydrides, or carbodiimides. *N*-Oxides react violently with carbodiimides such as DCC and EDC. This reaction has led to a number of industrial accidents where NMO is used as a solvent in the Lyocell process in cellulose manufacture. Residual NMO can therefore induce an autocatalytic decomposition if carbodiimides are used for acylation of cellulose.^40^ Nonclassical Polonovski reactions are initiated by metal cations and have been used for the dealkylation of tertiary amines to secondary amines.^41^

Aromatic *N*-oxides are typically more stable and cannot undergo the aforementioned rearrangements or eliminations. *N*-Oxides can also participate in a single electron transfer. Pyridine-*N*-oxide has been shown to have similar properties as the tyrosine/tyrosyl radical redox couple and can thus serve as an electron shuttle in artificial photosynthesis (Figure 3D).^42,43^ Along the same lines, polymeric *N*-oxides have been found to have special electron transport properties useful for the construction of semiconducting polymers^44^ and interlayer materials for organic solar cells.^45^ This special radical reactivity might also be the reason for the antioxidant properties, which have been suggested for several amphiphilic *N*-oxides used in cosmetic applications.^46,47^

## 3 Synthesis of *N*-Oxides

The synthesis of *N*-oxides by oxidation of tertiary amines is straightforward, yet attention to detail can be crucial for applications in medicinal chemistry. The aim of this section is to highlight the most relevant synthetic procedures addressing challenges and benefits. Comprehensive reviews are available for a more general overview of syntheses of *N*-oxides.^25,48−50^ As the purity of compounds for pharmaceutical research is of particular importance, this section also explores some qualitative and quantitative analyses of *N*-oxides.

**Figure 4:**
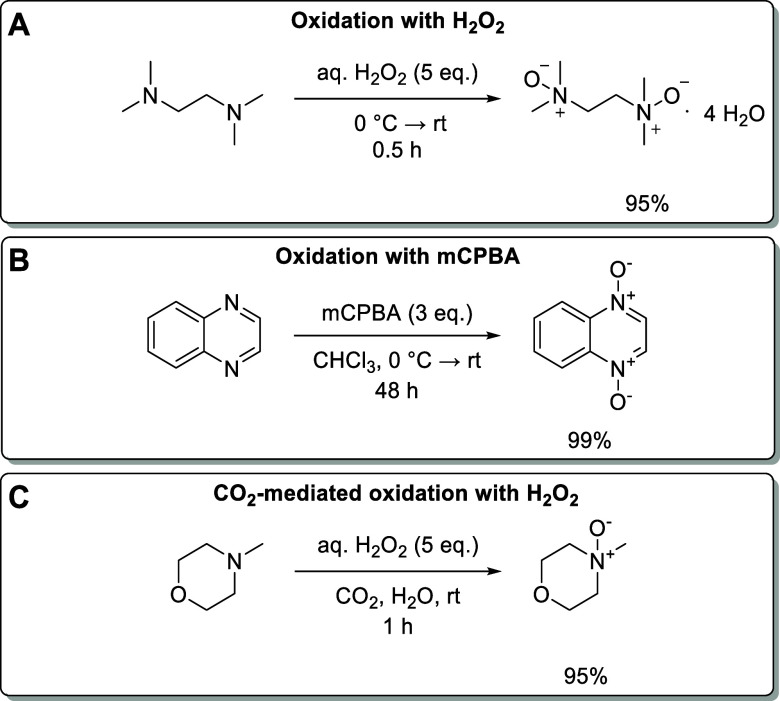
Examples for the synthesis of *N*-oxides: A) oxidation of *N,N,N*′*,N*′-tetramethylethane-1,2-diamine with H_2_O_2_;^64^ B) oxidation of quinoxaline with mCPBA;^65^ and C) CO_2_-mediated oxidation of *N*-methyl-morpholine-*N*-oxide (NMO) with H_2_O_2_.^66^

The oxidation of tertiary amines with molecular oxygen is often a sluggish reaction and requires harsh conditions (i.e., high oxygen pressure, elevated temperature).^51,52^ Other oxidizing agents such as H_2_O_2_ or peroxyacids are therefore typically preferred.^53^ The chemoselectivity of these reactions is determined by the basicity of the amine. A general trend is more basic amines are more readily oxidized by the electrophilic oxidants. H_2_O_2_ is particularly attractive in this context because it is a reasonably stable and cost-effective reagent that generates only H_2_O as a byproduct of oxidation. It allows the oxidation of most tertiary amines to the corresponding *N*-oxides with high atom economy on laboratory and industrial scales^39^ and is often used in metal-catalyzed protocols.^54−56^ The uncatalyzed oxidation with H_2_O_2_ is a relatively slow reaction, and a large excess of oxidant is often used to drive the reaction to completion (for an example, see Figure 4A).^37^ However, *N*-oxides form stable hydrogen bonds with H_2_O_2_, and it is therefore often difficult to remove excess oxidant. Notably, many studies ignore the analysis of residual H_2_O_2_ in *N*-oxide products, although it might cause safety concerns. In addition, H_2_O_2_ has similar properties compared to many *N*-oxides, such as oxidative and antibacterial activity.^16,57,58^ For applications of *N*-oxides as redox-active compounds in a pharmaceutical context, it is therefore imperative to confirm the absence of H_2_O_2_. Due to the oxidative properties of *N*-oxides, iodometric titration is not a reliable method for quantification of residual H_2_O_2_.^59^ In general, H_2_O_2_ can be detected via NMR spectroscopy,^60,61^ enzymatic colorimetric analysis (e.g., horseradish peroxidase assay),^62^ or chemical colorimetric analysis (e.g., titanium sulfate).^63^ For laboratory purposes, colorimetric peroxide test strips can confirm the absence of H_2_O_2_ (≤0.5 mg/L).

Residual H_2_O_2_ can be quenched by additives like sodium hypochlorite, sodium thiosulfate, sodium sulfite, activated carbon, Pt, MnO_2_, and Pd/C.^67,68^ In many cases, these additives are easily removed by filtration during work up. However, residual metal can be hard to separate from the final product due to the formation of complexes. The quenching additive might therefore also impact applications of *N*-oxides. This is particularly relevant for traces of transition metals. Mn(IV), for example, has been shown to oxidize aromatic *N*-oxides, leading to reactive radical species.^69^ H_2_O_2_ removal with activated carbon is therefore a preferable option to avoid metal impurities in the final products.

Various other electrophilic oxidants have been used for the oxidation of tertiary amines and aromatic amines to the corresponding *N*-oxides.^48^ Peroxyacids are a particularly useful alternative to H_2_O_2_ for the oxidation of tertiary amines, but their use is accompanied by a loss of functional group tolerance.^70^ While H_2_O_2_ poorly reacts with double bonds, carbonyls, or thioethers in the absence of a catalyst, peroxyacids convert these functional groups to epoxides, esters, and sulfones, respectively.^71,72^ The most commonly employed reagent on a laboratory scale is *meta*-chloroperbenzoic acid (mCPBA), and numerous synthetic procedures have been published (for an example, see Figure 4B).^73,74^ However, mCPBA is disadvantageous for reactions on an industrial scale due to its high cost and safety concerns. A workaround is the in situ generation of peroxyacids by oxidizing the corresponding carboxylic acids with H_2_O_2_ or other oxidants. A particularly attractive variant uses a mixture of H_2_O_2_ and CO_2_ as an oxidant. This CO_2_-mediated oxidation involves the formation of peroxymonocarbonate, which has a 400-fold higher second-order rate constant for the oxidation of aliphatic tertiary amines (Figure 4C) than H_2_O_2_ alone.^66^ Similar protocols use mixtures of H_2_O_2_ and acetonitrile.^75^ However, the latter protocol generates acetamide, which can be hard to remove from the reaction products.

The oxidation of pyridines and other heteroaromatic compounds to the corresponding *N*-oxides is typically achieved by the same methods as outlined above, and the reader is referred to the review literature for more detailed information.^76^

The oxidation progress can be monitored by the consumption of the amine using TLC. Dragendorff reagent is effective to visualize aliphatic *N*-oxide products on TLC plates.^77^*N*-Oxides can furthermore be analyzed by NMR spectroscopy. The introduction of oxygen leads to a downfield shift of neighboring methyl, methylene, or methine groups compared to the parent amine in the ^1^H- and ^13^C NMR spectra. In IR spectroscopy, the N^+^–O^–^ bond typically exhibits a prominent vibration band around 930 cm^–1^.^78^ Quantitative analysis of *N*-oxides is also possible via acid–base titration or the deoxygenation with phenylboronic acid.^79,80^ However, special attention must be paid to potential competitive reactions involving residual amine and residual oxidants such as H_2_O_2_.

## 4 Natural Products Containing *N*-Oxides

*N*-Oxides can be found in various natural sources including plants, microorganisms, and animals. Numerous *N*-oxides derived from alkaloids have been described with diverse biological activities, including antibiotic and cytotoxic effects.^81^

**Figure 5:**
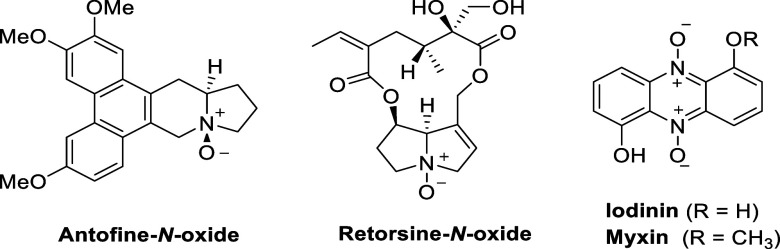
Structures of the indolizidine antofine-*N*-oxide, the pyrrolizidine retorsine-*N*-oxide, and the phenazines iodinin and myxin.

Indolizidine-*N*-oxide alkaloids are an interesting class of heterocyclic aromatic compounds that are found in many plants. Some of the isolated *N*-oxides have antimicrobial, antibacterial, antifungal, or antitumor properties.^82^ For example, antofine-*N*-oxide (Figure 5) is a cytotoxic indolizidine-*N*-oxide, which was isolated from *Cynanchum vincetoxicum*. Remarkably, antofine-*N*-oxide has different effects on cell lines derived from solid tumors and white blood cells. It inhibits the proliferation of brain, breast, and lung cancer cells and induces apoptosis in T-cell leukemia cells via TNFα signaling. An approximately 10-fold lower cytotoxicity of the compound in noncancerous fibroblasts suggests potential as an antitumor drug.^83^ A range of antofine derivatives (including many *N*-oxides) has been investigated with respect to their cytotoxicity. However, a clear trend in the SAR of these compounds can hardly be derived from these studies.^82^

Pyrrolizidine-*N*-oxides have typically lower acute and long-term toxicity than the corresponding tertiary amines and both usually coexist in plants.^4^ They are present in 3% of all flowering plants such as *Asteraceae*, *Boraginaceae*, and *Leguminosae*.^84^ They play a role in chemical ecology, as pyrrolizidine-*N*-oxides are reduced by enzymes in the liver and microbes of the gastrointestinal tract of herbivores. The resulting pyrrolizidine alkaloids have acute liver toxicity and cause the hepatic sinusoidal obstruction syndrome.^85^ Their long-term toxicity is most likely a result of the formation of protein and DNA adducts, which contribute to cancer development.^86^ Along these lines, *N*-oxides are often used by insects and plants as nontoxic storage compounds of otherwise toxic alkaloids containing tertiary amines against herbivores and predators. Due to their high polarity, these *N*-oxides are often unable to permeate membranes and can be stored in vesicles. On the other hand, some herbivores metabolize toxic tertiary amines to less toxic *N*-oxides, through enzymatic oxidation as a detoxification pathway.^4,87^ However, not all pyrrolizidine-*N*-oxides have a low toxicity. Retorsine-*N*-oxide, for example, has acute hepatotoxicity (Figure 5), although it is slightly less toxic than its parent amine retorsine.^88^

The phenazines iodinin and myxin (Figure 5) are well-known natural products with cytotoxic properties. Iodinin, first isolated in 1938 from *Brevibacterium iodinum*, has a selective cytotoxicity for leukemia cells.^89^ Myxin has been found to have potent broad-spectrum antimicrobial activity against several bacteria and fungi. Several modes of action have been described including DNA-intercalation, inhibition of topoisomerases, metal chelation, and production of reactive oxygen species. Under hypoxic conditions, the *N*-oxide groups are enzymatically reduced and form hydroxyl radicals.^90^ The discovery of this latter mechanism was the starting point for the development of hypoxia-selective drugs like tirapazamine (vide infra) and many other compounds with anti-infective or anticancer activity.^91,92^

*N*-Oxides are also present in human metabolism in the form of TMAO, a metabolite of trimethylamine that is formed in the liver by hepatic flavin monooxygenases (FMO1 and FMO3). Trimethylamine itself derives from nutrient substrates produced by the metabolism of phosphatidylcholine/choline, carnitine, betaine, dimethylglycine, and ergothioneine by intestinal microflora in the colon.^93^ Elevated systemic concentrations of TMAO have been associated with a number of cardiovascular diseases.^94^ In addition, TMAO has been implicated in neurodegenerative diseases like Alzheimer’s disease. It has also been suggested that the gut microbial-derived metabolite trimethylamine and TMAO might have an important function for the communication via the gut–brain axis with wide consequences for cerebrovascular and cognitive function.^95,96^

## 5 Drugs Containing *N*-Oxide Groups

**Figure 6:**
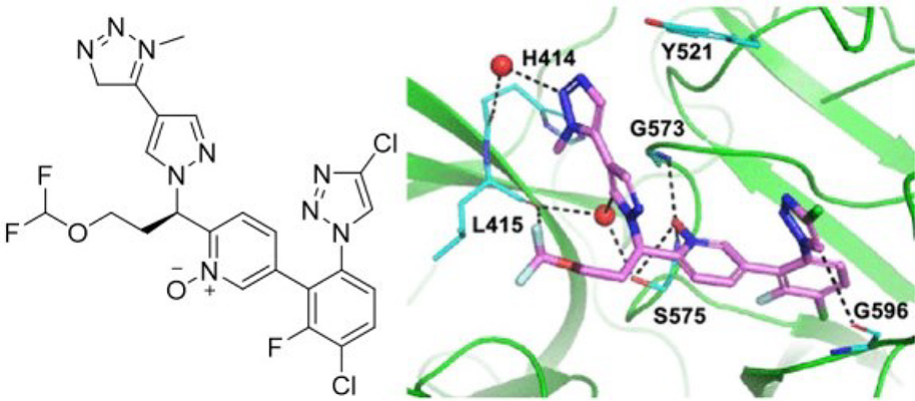
Structure of a pyridine-*N*-oxide inhibitor and the docking pose with protease factor XIa. Adapted with permission from ref (103). Copyright 2022, American Chemical Society.

A number of drugs have an *N*-oxide functionality. In this section, we discuss the relevance of this moiety for the pharmacological properties and applications of these compounds. The N–O group is often critical for the biological activity of these compounds. It can serve as a mimic of nitric oxide (NO), leading to NO-like effects such as vasodilation.^3^ Several furoxane derivatives (1,2,5-oxadiazole-*N*-oxides) are NO-donors in vivo and are thus interesting candidates for drugs affecting vasodilation, platelet aggregation, or other pharmaceutically interesting processes mediated by NO.^97,98^ The cytotoxic effects of NO are used for the development of anti-infectives and anticancer compounds such as redox-activated furoxanes.^99^ However, the mode of action of several furoxanes and related indazole-*N*-oxides is still debated and might include different components.^100^ Some benzofuroxanes are active against *Leishmania* infections. In this case, a dual action of the *N*-oxide drug has been proposed involving NO generation and inhibition of a *Leishmania* cysteine protease.^101^ In contrast, no NO generation was found for a benzofuroxane with potent activity against *Mycobacterium tuberculosis*.^102^ Due to their strong hydrogen bonds, *N*-oxides can also be used as bioisosteric replacements of hydrogen bond acceptors and have been used in the design of factor XIa inhibitors. Factor XIa is a blood coagulation protease, and a pyridine-*N*-oxide derivative has been shown to interact favorably with the oxyanion binding site in the enzyme via its pyridine *N*-oxide core (Figure 6).^103^

**Figure 7:**
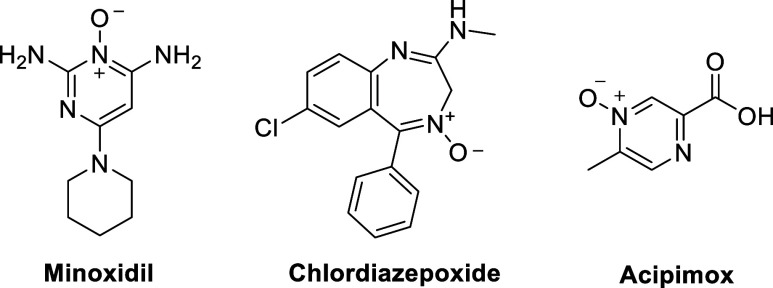
Heterocyclic *N*-oxide scaffolds in drugs: some heterocyclic *N*-oxides release NO after reductive activation. Structures of minoxidil, chlordiazepoxide, and acipimox.

Minoxidil (Figure 7) is a prodrug that was originally developed to treat high blood pressure. However, it is commonly used to treat hair loss, particularly in androgenetic alopecia, also known as hereditary hair loss. Minoxidil sulfate is the active metabolite formed enzymatically via a sulfotransferase in vivo. The active metabolite is a potassium channel opener and leads to vasodilation. The exact mechanism of action is not fully understood. However, as a NO mimic, it dilates blood vessels, leading to improved blood flow to the scalp. This could provide more oxygen, nutrients, and hormones to the hair follicles, which could stimulate hair growth.^3^

Chlordiazepoxide (Figure 7) is the first benzodiazepine that has been discovered and belongs to the group of psychotropic drugs. Chlordiazepoxide is used for the short-term treatment of anxiety and alcohol abuse. It also showed anticonvulsant properties. Chlordiazepoxide works as an allosteric modulator for the GABA_A_ receptor. Its main effect is based on influencing the central nervous system, in particular by increasing the inhibitory effect of the neurotransmitter gamma-aminobutyric acid (GABA).^104^ GABA molecules bind to the orthosteric agonist pockets while benzodiazepines typically occupy the canonical binding site at the α1+/γ2– interface of the human synaptic GABA_A_ receptor.^105^ Chlordiazepoxide and other CNS-active drugs involving the polar *N*-oxide motif, have stimulated studies on the permeability of these *N*-oxides through the BBB. However, despite numerous efforts to investigate this aspect, the ability of *N*-oxides to cross the BBB remains a subject of controversial debate. The reversible redox metabolism of *N*-oxides and corresponding amines as well as the presence of the responsive enzymes in the periphery *and* the brain complicate these investigations. Drawing definitive conclusions from drug/metabolite concentrations in cerebrospinal fluid and plasma is therefore difficult, even for simple compounds like TMAO.^95,96,106^

Acipimox (Figure 7) is a medication for hyperlipidemic patients that are unresponsive to alternative therapeutic approaches. It inhibits lipolysis and restricts the flow of free fatty acids to the liver via binding to the G-protein-coupled receptor HCAR2 on adipocytes.^107^ This leads to a reduction in the precursor pool size of the very low-density lipoprotein (VLDL)-triglyceride. It inhibits VLDL synthesis, and consequently lowers plasma triglyceride levels while elevating high-density lipoprotein (HDL).^108^

**Figure 8:**
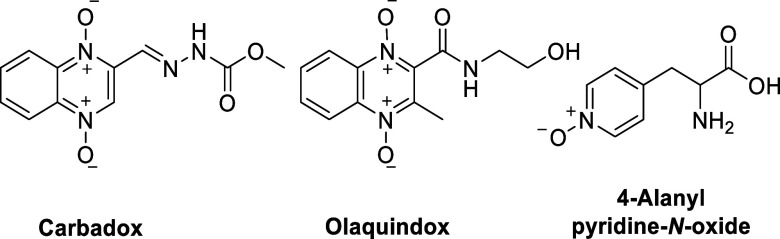
Structures of the antimicrobial quinoxalines carbadox and olaquindox and 4-alanylpyridine-*N*-oxide.

Carbadox and olaquindox (Figure 8) are structural analogues of the natural products iodinin and myxin. They have been used as antimicrobial agents in veterinary medicine as feed additives to promote growth. Both have an antibacterial effect by inhibiting the growth of pathogenic bacteria in the digestive tract of animals. In addition, olaquindox can influence metabolism, leading to a more efficient conversion of nutrients into body weight in animals.^109^ Olaquindox has also been reported to cause photoallergy.^110^ The mode of action of both drugs is not fully understood. However, reductive activation and the formation of reactive radical species are likely important contributors to antimicrobial activity. Due to concerns about residues and antibiotic resistance, its use has been restricted or banned in some regions.^111^ It is also notable that antibacterial activity has been reported for 4-alanylpyridine-*N*-oxide^112^ and 4-nitropyridine-*N*-oxide (Figure 8).^113^ The latter is a quorum sensing inhibitor in *Pseudomonas aeruginosa*, most likely through binding to LasR.^114^ Many Gram-negative bacteria, such as *P. aeruginosa*, communicate via autoinducers of the *N*-acylhomoserine lactone-type.^115^ Such communication is termed quorum sensing and is critical for the pathogenicity and resistance development in biofilms. Quorum sensing inhibitors can therefore attenuate the pathogenicity of *P. aeruginosa*. LasR is a key receptor for bacterial autoinducers and is therefore an interesting target for anti-infective drugs. 4-Alanylpyridine-*N*-oxide might act via similar mechanisms. In addition, a reductive enzymatic conversion of the *N*-oxide was demonstrated at least in *E. coli*.^112^ Even simple pyridine-*N*-oxides might thus be activated by enzymatic reduction similar to the phenazine and furoxane derivatives mentioned above. Some studies have also reported antimicrobial activity of long-chained alkylamine-*N*-oxides^58,116,117^ and some oligoamine-*N*-oxides.^118^ The latter have MICs in the low millimolar range against *S. aureus* and *C. albicans* but are inactive against *E. coli*. The mode of action has not been elucidated in these cases, and it is not clear if the antimicrobial activity is caused by the amphiphilic structure of the compounds or other mechanisms.

**Figure 9:**
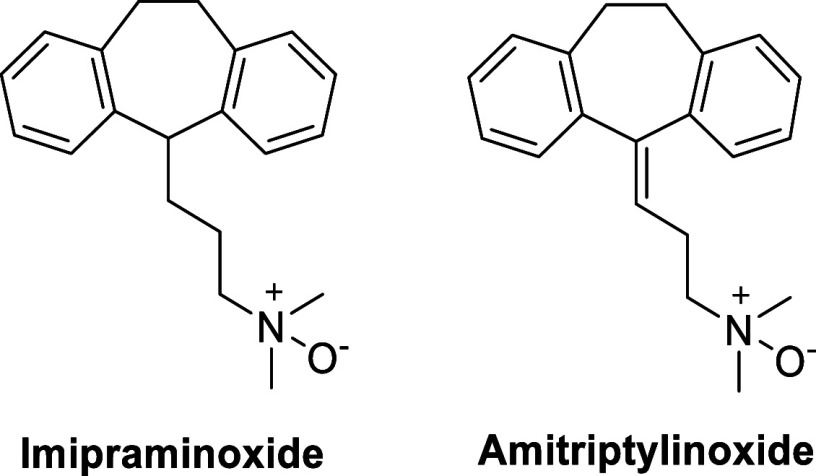
Structures of the benzepine prodrugs imipraminoxide and amitriptylinoxide. Both are also oxidative metabolites of the parent drugs imipramine and amitriptyline.

As mentioned above, *N*-oxides are important water-soluble metabolites of tertiary amines. On the other hand, *N*-oxides can also be reduced to the parent amines in vivo. Imipraminoxide and amitriptylinoxide (Figure 9), for example, which are tricyclic antidepressants, are considered prodrugs of the corresponding tertiary amines imipramine and amitriptyline. Imipramine and amitriptyline act as serotonin-norepinephrine reuptake inhibitors.^119^ Imipraminoxide and amitriptylinoxide are also metabolites of imipramine and amitriptyline. Amitriptyline, for example, is enzymatically oxidized to amitriptylinoxide by a flavin-containing monooxygenase (FMO).^120^ Both *N*-oxides have a faster onset of action and fewer side effects (reduced drowsiness, sedation, and anticholinergic symptoms such as dry mouth, sweating and dizziness) than the corresponding tertiary amines.^121^ The redox properties of several *N*-oxides derived from antipsychotic drugs based on benzepine scaffolds have been evaluated by chemical methods.^122^ However, it is unclear whether these compounds contribute to oxidative tissue damage of the parent drugs in vivo.

**Figure 10:**
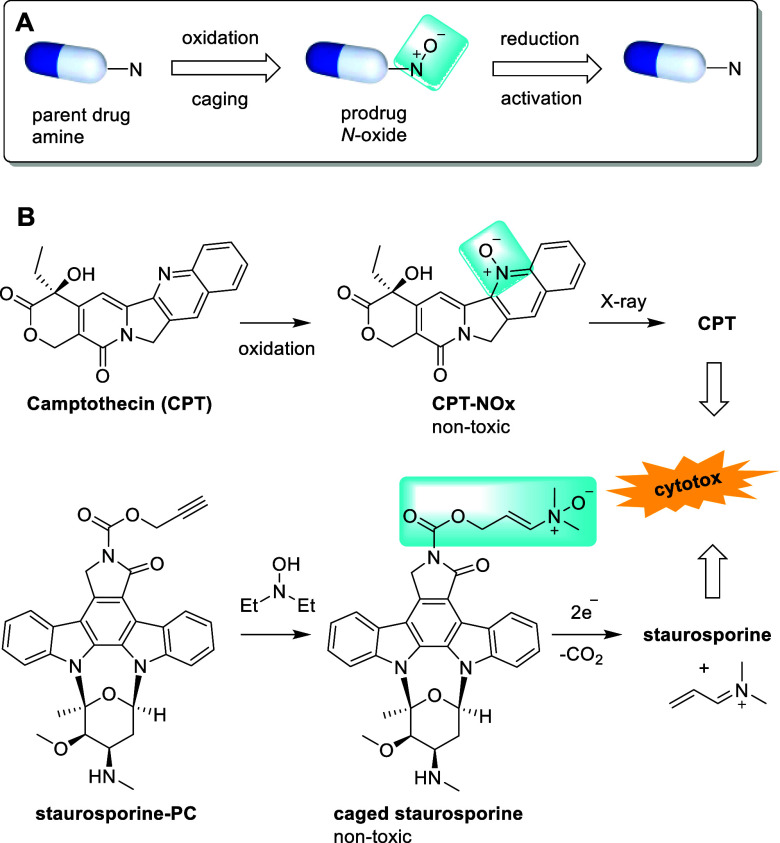
*N*-Oxides can be used for the caging of cytotoxic compounds. A) Schematic drawing of the prodrug concept. B) Caging of the cytotoxic compounds camptothecin and staurosporin via the corresponding less toxic *N*-oxides and their reductive activation in vivo.

Certain *N*-oxides can be reduced to their parent amines in vivo either enzymatically or by hydrated electrons (e^–^_aq_) generated through an X-ray treatment. The related prodrug concept for cancer therapy is depicted in Figure 10A. It is particularly appealing when highly cytotoxic compounds are caged as nonactive *N*-oxide prodrugs, which can selectively be activated at the tumor site. Conjugated *N*-oxides derived from anilines or heteroaromatics have sufficiently low HOMO/LUMO energy gaps to be susceptible to reduction by e^–^_aq_. Camptothecin (CPT, Figure 10B) is a potent inhibitor of topoisomerase I and bears a pyridine nitrogen. It can therefore be caged as the corresponding nontoxic *N*-oxide (CPT-NOx) and selectively be activated by X-ray treatment. This led to significant reduction in tumor growth in a mouse model.^7^ The prodrug concept was demonstrated to be of broad applicability to drugs containing nitrogen heteroaromatic moieties or anilines. Cytotoxic drugs in which alkylamines or amides are present can be caged with enamine-*N*-oxides. The concept has a broad scope because the *N*-oxide cages are formed via a retro-Cope reaction from alkynes and hydroxylamines. An example is depicted in Figure 10B. The caged enamine-*N*-oxide of the cytotoxic drug staurosporine was prepared from staurosporine propargylcarbamate (staurosporine-PC). Activation of the prodrug occurs via enzymatic reduction in hypoxic tumor tissue.^123^ This process releases the active drug from the caged conjugate and generates a reactive electrophile (Michael acceptor) from the enamine-*N*-oxide. This Michael acceptor might add to the cytotoxicity of the released drug. It can also be used for hypoxia-triggered tumor labeling and thus for theranostic approaches.

**Figure 11:**
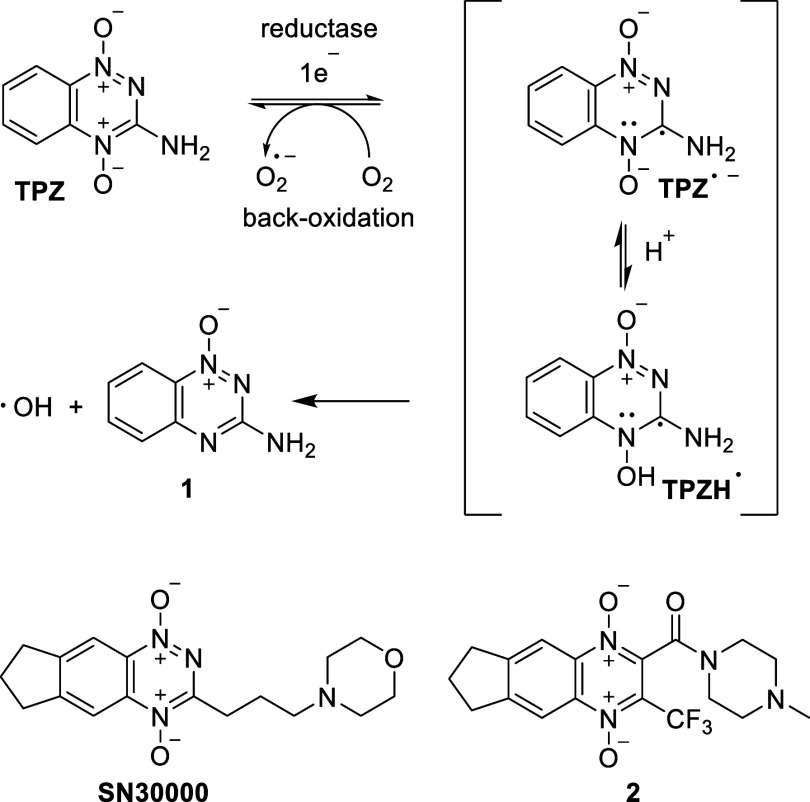
Hypoxia-activated prodrugs (HAPs): mechanism of the enzymatic activation of tirapazamine (TPZ) under hypoxic conditions and structures of second-generation derivatives SN30000 and **2**.

Hypoxic conditions are favorable for reductive biotransformations of *N*-oxides, which has been investigated for the conversion of the pyrrolizidine alkaloid indicine-*N*-oxide to indicine. Notably, carbon monoxide is an inhibitor of microsomal indicine-*N*-oxide reduction, implying the involvement of a cytochrome P450 oxidoreductase in this process.^124^ The exact mechanism of the reduction has not been fully understood, but it was found that several cytochrome P450 enzymes catalyze this reaction.^120^ This reductive activation of *N*-oxides under hypoxic conditions has been exploited for cancer treatment through the use of hypoxia-activated prodrugs (HAPs).^125^ Hypoxia is a common phenomenon observed in solid tumors. It is defined by low oxygen levels due to a poor blood supply. Hypoxia contributes to tumor progression, metastasis, and resistance to radiotherapy and chemotherapy.^126^ The typical activation mechanism of HAPs involves the action of reductases creating a reduced and reactive derivative of the prodrug.^127^ A prototype is tirapazamine (TPZ, Figure 11), first reported in 1986.^128^ It induces oxidative damage in hypoxic tissue through one-electron reduction, primarily facilitated by cytochrome P450 enzymes. The mechanism has been intensively investigated and is believed to proceed via a one-electron reduction of TPZ to the TPZ^•**–**^ radical anion.^129^ After protonation of TPZ^•**–**^ (p*K*_a_ ∼ 6) to TPZH^•^, a hydroxyl radical and the final deoxygenated metabolite **1** are formed. The generated hydroxyl radicals are believed to cause DNA double-strand breaks and chromosome aberrations, which are particularly challenging to repair.^129^ The antitumor effects include cell cycle arrest, apoptosis, and downregulation of HIF-1α, CA-IX, and VEGF expression.^130^ HAPs are particularly promising cancer therapeutics in combination with other cytostatic drugs, radioimmunotherapy, and hyperthermia. However, clinical trials with TPZ revealed different therapeutic success. Some demonstrated encouraging antineoplastic efficacy and tolerable toxicity, while others showed only a limited benefit on survival or significant adverse effects.^8^ The low bioavailability of TPZ has recently been addressed with the conjugation to a pH-responsive nanocarrier.^131^ TPZ and several other benzotriazine-di-*N*-oxides also have antimicrobial activity against various clinically relevant pathogens.^132^ Particularly interesting is their activity against *Mycobacterium tuberculosis* (Mtb). It differs from antitumor activity in that most benzotriazine-di-*N*-oxides tested are active against replicating Mtb *and* hypoxia-induced nonreplicating Mtb. This finding suggests that the reductases in Mtb responsible for activating the drug are expressed during both of the replicating and nonreplicating metabolic states and that back-oxidation to the parent compound under normoxic conditions does not significantly affect antitubercular activity.^133^

SN30000 is a second-generation benzotriazine-*N*-oxide HAP and a modified analogue of TPZ. Currently in preclinical research, SN30000 has a similar mechanism of action compared to TPZ but showed superior antineoplastic effects and hypoxia selectivity.^134^ Studies highlight its heightened activity on tumor spheroids, leading to significant tumor growth delay when combined with radiation.^135^ In addition, SN30000, when used with gemcitabine, effectively inhibits the proliferation of reoxygenated tumor cells.^136^ The binding of EF5 may serve as a promising biomarker for hypoxia stratification and the assessment of SN30000 treatment response.^137^ It was found that the substitution pattern of the heterocyclic scaffold influences the back-oxidation of the intermediately formed radical anion (e.g., TPZ^•–^) and thus the selective hypoxic release of hydroxyl radicals. Trifluoromethyl-substituted derivatives like **2** also showed significant cytotoxicity under aerobic conditions.^138^

**Figure 12:**
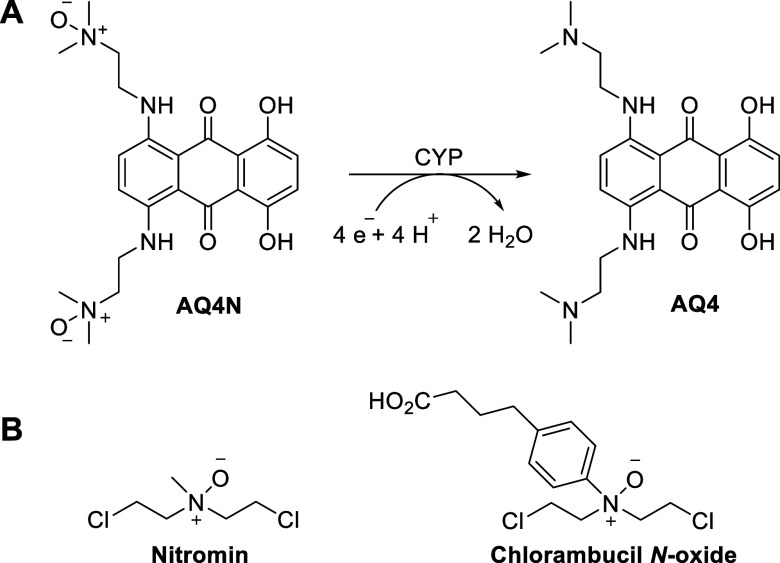
Aliphatic *N*-oxides as prodrugs for use in cytotoxic drugs. A) Enzymatic activation of AQ4N (banoxantrone) to cytotoxic AQ4 by CYP oxidoreductases. B) *N*-Oxide derivatives of nitrogen mustards.

AQ4N (banoxantrone, Figure 12A) is an aliphatic *N*-oxide first reported in 1993.^139^ Under hypoxic conditions, AQ4N undergoes a two-electron reduction mediated by CYP, producing AQ4, a potent topoisomerase II inhibitor with high cytotoxicity.^140^ Phase I clinical studies have revealed good antineoplastic effects in combination with radiotherapy or chemotherapy. AQ4N showed antitumor effects in various cancer models, including pancreatic, bladder, lung, prostate, and gliosarcoma. However, the development was discontinued due to unfavorable outcomes in clinical phase II studies.^141^ AQ4N is only a recent example of a cytotoxic drug that is targeted to hypoxic tumor tissue with an aliphatic *N*-oxide. The approach has also been successful with other cytotoxic compounds such as the nitrogen mustard derivatives nitromin^142^ and chlorambucil-*N*-oxide^143^ (Figure 12B). A similar two-electron enzymatic reduction of indolone *N*-oxides has been proposed to occur in red blood cells which might be important for the pharmacokinetics of several redox-active *N*-oxides.^144^

**Figure 13:**
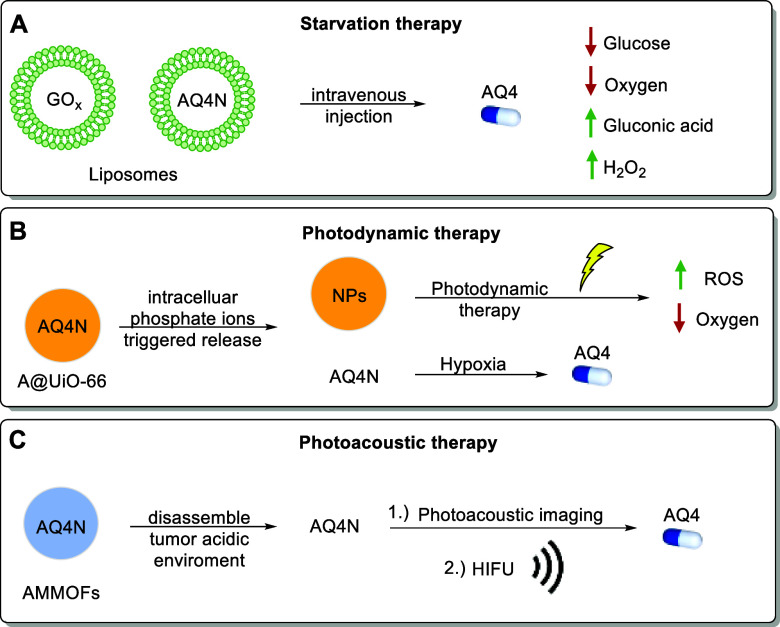
AQ4N combination therapies: A) starvation therapy, B) photodynamic therapy, and C) photoacoustic therapy.

The concept of hypoxia activation is appealing, but there is currently no approved HAP available, and all candidates failed in clinical trials.^145^ One reason might be the difficult delivery of these compounds to the hypoxic target cells. These are typically located in tissue distant from functional blood vessels. In addition, patient stratification based on hypoxia status is expensive and therefore limiting clinical trial participation.^127^ Mutagenicity has also been reported for some heterocyclic *N*-oxides, although it is not a general phenomenon associated with these compounds and is strongly dependent on the substitution patterns.^146^ The concept of hypoxia activation continues to stimulate new therapeutic strategies involving HAPs. AQ4N, for example, has been used recently in combination with photoacoustic therapy, photodynamic therapy, and starvation therapy (Figure 13).^147−149^

Starvation therapy using liposomes loaded with glucose oxidase (GOx) is proposed to cut off the tumor’s energy supply and slow its growth (Figure 13A). Intravenous injection of these liposomes leads to effective tumor retention, depleting glucose and oxygen within the tumor. This process produces cytotoxic H_2_O_2_ and enhances hypoxia, as shown by noninvasive in vivo imaging. Combining starvation therapy with liposomes carrying AQ4N results in synergistically enhanced tumor growth inhibition in a mouse model.^147^

In photodynamic therapy (PDT, Figure 13B), severe hypoxia often limits effectiveness due to oxygen consumption. A novel approach uses azido-/photosensitizer-terminated UiO-66 nanoscale metal–organic frameworks (A@UiO-66 NPs) as nanocarriers for the bioreductive prodrug AQ4N. These nanocarriers effectively shield AQ4N, preserving its stability. A dense PEG layer, introduced by azide–alkyne cycloaddition, enhances their dispersion and improves the therapeutic performance. The oxygen-depleting PDT process aggravates hypoxia, activating AQ4N’s selective activation for synergistic therapy. Both in vitro and in vivo studies demonstrated enhanced therapeutic efficacy of AQ4N with negligible systemic toxicity, making this hybrid nanomedicine a valuable candidate for cancer therapy.^148^

Photoacoustic (Figure 13C) imaging is promising for monitoring high-intensity focused ultrasound (HIFU) surgery. However, a common drawback is limited tissue penetration. A new approach with AQ4N was introduced with a metal–organic framework nanosystem, combining AQ4N and Mn(II) (AMMOFs). This system enhances signal penetration through deep tissue, guiding HIFU surgery more accurately. In the hypoxic tumor environment, AQ4N is activated to eliminate residual hypoxic tumor cells.^149^

In addition, hypoxia-induced reduction of polymeric *N*-oxides has been shown recently to be valuable for drug delivery (vide infra).^14^

**Figure 14:**
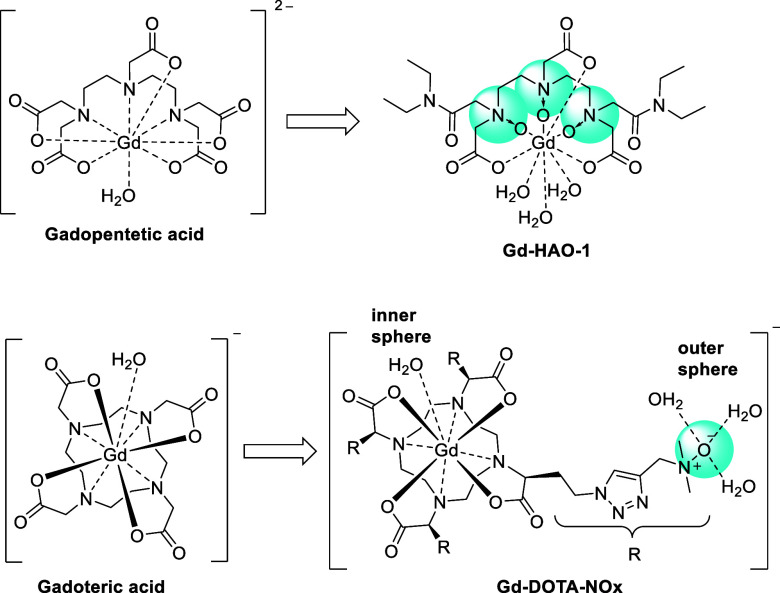
Gadopentetic acid and gadoteric acid are common Gd-based contrast agents for MRI. The relaxivity of these reagents was increased with the introduction of *N*-oxide functionalities in derivatives Gd-HAO-1 and Gd-DOTA-NOx.

The properties of *N*-oxides have also been used to improve Gd-based contrast agents used for magnetic resonance imaging (MRI). HAO-1 (Figure 14), for example, is an *N*-oxide derivative of the chelator diethylenetriaminepentaacetate (DTPA). In the form of a Gd-complex (gadopentetic acid, Magnevist), this compound has been used frequently as a MRI contrast agent until 2017.^150^ Gd-HAO-1 has an increased hydration number (q = 3) compared to gadopentetic acid (q = 1), leading to a significantly increased relaxivity and thus higher potential sensitivity in MRI.^151^ Typically, an increase in the hydration number comes with a decreased complex stability. However, *N*-oxides form more stable metal complexes than their parent amines, and complexes like Gd-HAO-1 combine; therefore, a high hydration number with reasonable stability.^152^ The good complexation properties of *N*-oxides have also been used by other researchers to assemble new Gd-based contrast agents for MRI.^153,154^ In addition, *N*-oxides are strongly hydrated in an aqueous solution. Like other zwitterions, they can thus be used for conjugation to drugs to improve their solubility, increase their Stokes radius, and reduce unwanted tissue or protein binding.^155,156^ Along these lines, the decoration of gadoteric acid (Dotarem) with four *N*-oxide functionalities (not involved in metal binding) led to the development of Gd-DOTA-NOx with almost three times higher relaxivity.^157^

## 6 Polymeric *N*-Oxides for Drug Conjugation and Surface Engineering

**Figure 15:**
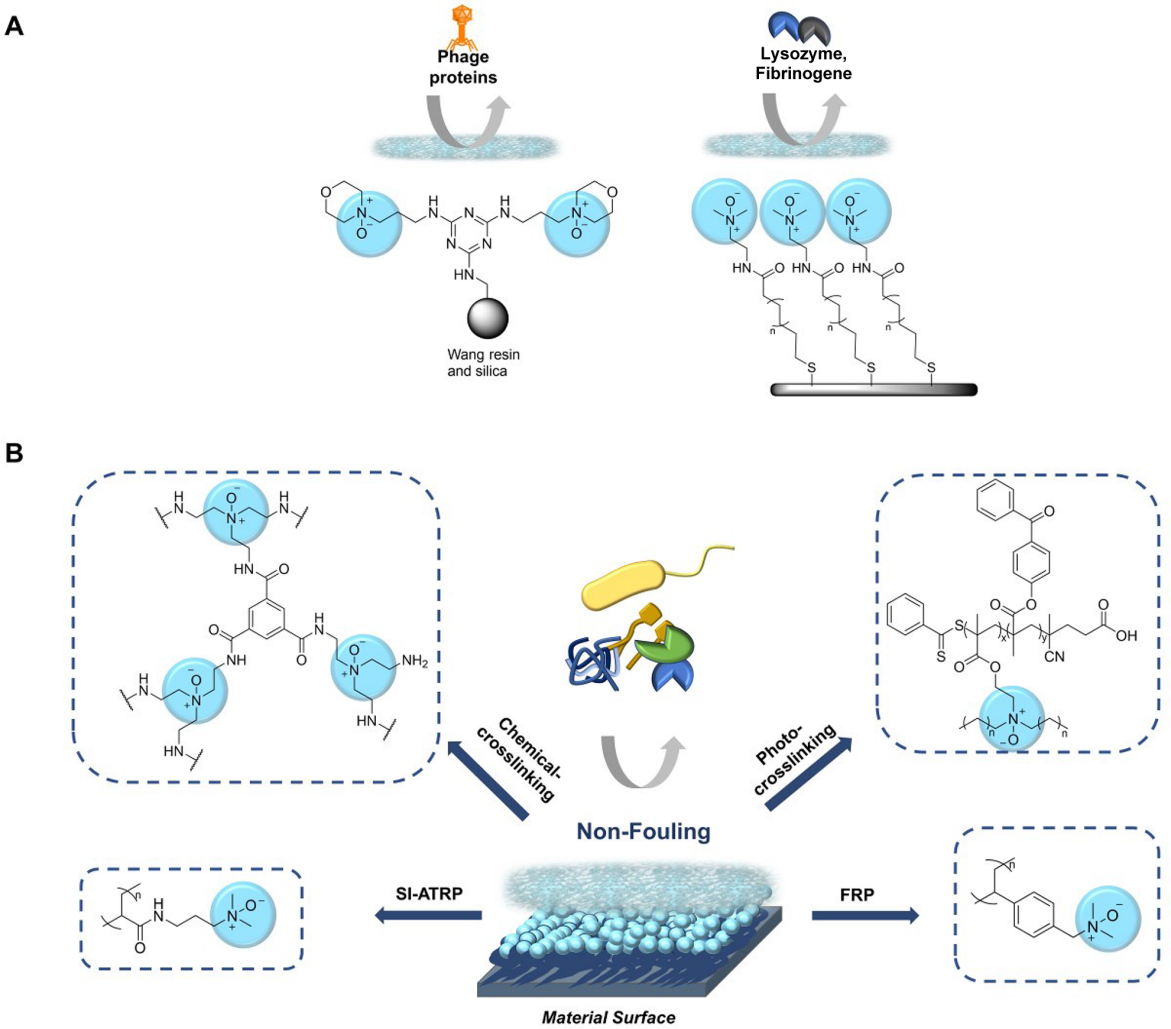
Materials loaded with *N*-oxides are highly hydrated and nonadhesive for biomolecules or microorganisms. A) First examples of immobilized *N*-oxides on silica particles, Wang resin, and gold. B) Common approaches to polymeric *N*-oxides. Abbreviations: surface-initiated atom transfer radical polymerization (SI-ATRP), free radical polymerization (FRP).

*N*-Oxides are easy to prepare, typically nontoxic and have a number of favorable physical and chemical properties to meet critical challenges in applications of biomaterials or the formulation of drugs. As mentioned above, *N*-oxides are kosmotropes and form strong hydrogen bonds. Once assembled on surfaces or in oligo- or polymeric structures, they are highly hydrated. The resulting interfaces do not bind to proteins or other biomolecules. They are thus nonimmunogenic and have excellent blood compatibility, which is often described with the term “stealth property”. Polymeric *N*-oxides thus resemble a lot of properties of other hydrophilic polymers (e.g., PEG or polysulfobetaines) used for drug or surface conjugation in the biomedical field.^17,158^ The conjugation of *N*-oxides to particles and bulk materials to generate stealth surfaces has been pioneered by Marsh and co-workers with the immobilization of NMO to silica particles and Wang resin (Figure 15A).^12^ It was later extended to self-assembled monolayers of *N*-oxides on gold surfaces.^159^ Both approaches lead to stealth properties of the resulting materials with low adsorption of fibrinogen, lysozyme, and a protein mixture of a phage protein library.

Polymeric *N*-oxides have been known for a while and used as oxidants in organic synthesis,^160^ as interlayer materials for solar cells^45,161^ and for application in nonlinear optics.^162^ In a pharmaceutical context, polyvinylpyridine-*N*-oxide has been explored for the treatment of silicosis, a lung fibrosis induced by quartz fiber^163,164^ and the negative effects of coal dust.^165^ The use of polymeric *N*-oxides as stealth materials has recently been described and has found numerous applications in the past few years (Figure 15B). Polyacrylamide-derived *N*-oxides have been demonstrated to have low protein binding and low immunogenicity as a polymer hydrogel and also after surface-initiated grafting of the polymeric *N*-oxide from gold surfaces^11^ and membranes.^166^ Both protocols involve a bromoisobutyrate initiator for radical polymerization, which was immobilized either via a thiol functionality on gold or via a dopamine derivative on membranes. Grafting of polystyrene-derived *N*-oxides from plastics has also been reported to give stealth surfaces with nonadhesive properties for microorganisms.^16,167^ An alternative coating method for plastics is spin coating with subsequent cross-linking, which has been achieved either photochemically^168^ or with a chemical cross-linker.^169^ In each case, nonadhesive properties for proteins, blood platelets, and microorganisms were demonstrated, and the resulting materials are therefore highly attractive for application as fouling-resistant membranes and biomedical devices.^170^ The properties of polymeric *N*-oxides thus follow those of other polymeric zwitterions such as sulfobetaines, phosphobetaines, or carboxybetaines in many aspects.^17,171,172^ However, a particularly interesting feature of polymeric *N*-oxides is the low salt dependency of surface hydration.^173^ This superior resistance to salt effects compared to other zwitterionic polymers is due to the shorter distance between the positive and negative charges in *N*-oxides.^174^ It makes polymeric *N*-oxides interesting for applications not only in the biomedical field but also in (salt)water purification. The latter has been demonstrated with the preparation of superwetting membranes which can be used for the separation of oil–water emulsions^175^ and textile wastewater dye removal.^176−178^ A possible drawback of polymeric *N*-oxides for applications in healthcare is their limited thermal stability, which was mentioned in Chapter 2. Decomposition reactions typically start at around 120–150 °C. *N*-Oxides are therefore less thermostable than other zwitterions.^179^ They might thus not be compatible with common standard sterilization protocols.

The applications of polymeric *N*-oxides as inert stealth materials imply a lack of reactivity in biological media. However, biological activities were recognized for some polymeric *N*-oxides. A modification of polydopamine coassembled with aminopropyl-dimethylamine-*N*-oxide showed an antibacterial effect against *S. aureus*. The authors did not reveal the molecular mechanisms but attributed the activity to a membrane disruptive effect of protonated *N*-oxides (a so-called contact-activity)^180,181^ in a locally acidic environment.^182^ Antibacterial properties were also found for a polystyrene-derived *N*-oxide.^16^ In this case, radical formation was detected by spin traps and the ESR. A contact-active mechanism of polycationic surfaces derived from protonated *N*-oxide was found to be unlikely because the surface potential of the poly-*N*-oxide was almost neutral even at pH 4. Radical reactivity also aligns with the previously described bioactivity for antibacterial *N*-oxides of low molecular weight. These findings underline that the *N*-oxide reactivity depends on the chemical structure and the biological media of application.

**Figure 16:**
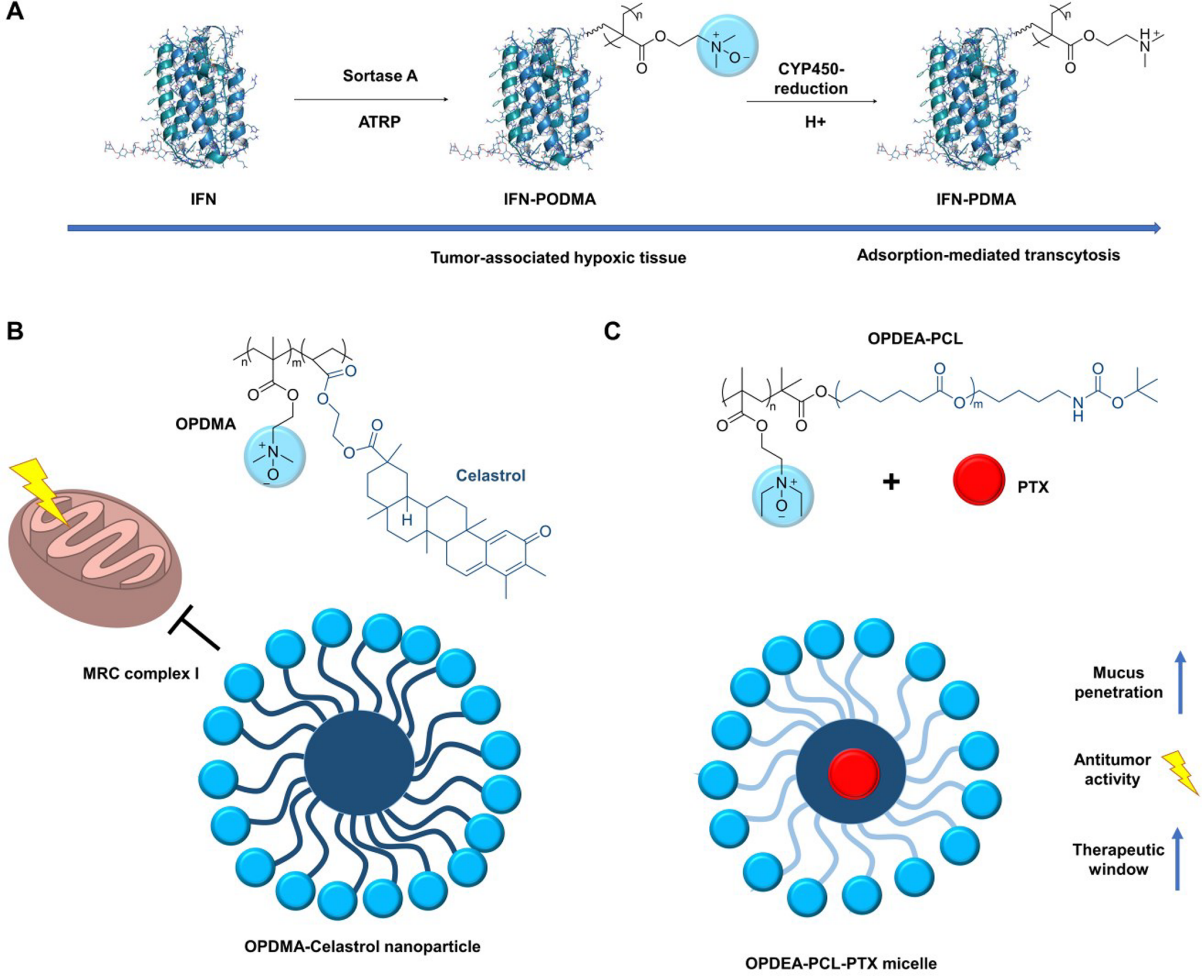
Protein conjugation and drug targeting through polymeric *N*-oxides. A) Enzymatic ligation of a bromoisobutyrate to IFN and subsequent SI-ATRP gave IFN-PODMA (poly(2-(*N*-oxide-*N,N*′-dimethylamino)-2-ethyl)methacrylate). The conjugate was enzymatically reduced to the corresponding polyamine IFN-PDMA (poly(2-(*N, N*′-dimethylamino)-2-ethyl methacrylate)) in hypoxic tumor tissue, allowing adsorption-mediated transcytosis. B) Micelles formed from polymeric *N*-oxide/ε-caprolactone copolymers penetrate the mucus and therefore allow oral delivery of drugs. Orally administered *N*-oxide micelles loaded with paclitaxel (PTX) therefore have stronger antitumor activity and a more favorable therapeutic window compared to PTX alone. C) Micelles of polymeric *N*-oxides accumulate in mitochondria. This feature is useful for the targeting of cytotoxic drugs acting on the mitochondrial respiratory chain such as the natural triterpenoid celastrol.

The conjugation of polymeric *N*-oxides to drugs/proteins allows a fine-tuning of their pharmacokinetic profiles and can improve solubility, increase blood half life, and decrease toxicity or immunogenicity as first demonstrated by conjugation of a polyacrylamide-derived *N*-oxide to uricase in a mouse model.^11^ The properties of the polymeric *N*-oxide conjugate were found to be comparable to those of the standard PEGylated analogues. However, in cancer therapy, PEGylation compromises the tumor penetration of drugs. Unlike PEG, *N*-oxides can be used not only to improve solubility, blood circulation time, and immunogenicity but also for tissue targeting because they are typically inert in aerobic conditions. Yet they can be enzymatically reduced in hypoxic tissue (vide supra). Following these principles, conjugates of a polymeric *N-*oxide and interferon alpha (IFN) showed an improved antitumor efficacy in mice compared to a PEGylated IFN (Figure 16A).^14^ The conjugates were obtained with a sortase-A-mediated ligation of a peptidic bromoisobutyrate to IFN and a subsequent atom transfer radical polymerization with acrylate-*N*-oxide. Once the conjugates reach hypoxic tumor tissue, they are enzymatically reduced to the corresponding polyamines, allowing an adsorption-mediated transcytosis. An improved tumor uptake of a small molecule conjugate to polymeric *N*-oxide has also been demonstrated with a camptothecin ester.^183^ Shen and co-workers have found that a polymethacrylate-derived *N*-oxide accumulated in the mitochondria of mouse embryonic fibroblast cells.^184^ This feature is useful for the targeting of cytotoxic drugs acting on the mitochondrial respiratory chain. The natural triterpenoid celastrol acts most likely on mitochondrial respiratory chain (MRC) complex I and induces cell apoptosis via ROS-dependent mitochondrial pathways. However, its poor water solubility, short plasma half-life, and high systemic toxicity impede its clinical use. The conjugation to a polymeric *N*-oxide leads to significantly improved therapeutic effects and decreased systemic toxicity compared to free celastrol (Figure 16B).^185^

The mitochondria targeting with *N*-oxides was also confirmed with a conjugate of dichloroacetate as an inhibitor of pyruvate dehydrogenase kinase 1 (PDHK1). The *N*-oxide conjugate induced mitochondrial oxidative stress through the inhibition of PDHK1, resulting in immunogenic pyroptosis in osteosarcoma cell lines.^186^ In addition, it has been shown that micelles formed from polymeric *N*-oxide/ε-caprolactone copolymers can penetrate the mucus and therefore allow the oral delivery of drugs. This was demonstrated in a proof-of-concept study for *N*-oxide-derived micelles loaded with paclitaxel (PTX, Figure 16C). Orally administered *N*-oxide micelles had stronger antitumor activity and a more favorable therapeutic window compared to PTX alone and a PEGylated PTX derivative.^187^

## 7 Summary and Perspective

The *N*-oxide group has a number of remarkable properties relevant for drug development, drug metabolism, and the design of materials for biomedical applications. Besides their well-known role as metabolites of tertiary amines, *N*-oxides are increasingly introduced into drug structures. Due to their high polarity and strong binding to water molecules, they typically improve the water solubility and decrease the membrane permeability of drugs. They might also be used as complex ligands for metals in pharmaceutically relevant chelators or to increase the Stokes radius and the stealth properties of contrast agents for MRI. The latter effect might also be advantageous for other diagnostic tracers, where good water solubility and stealth properties are often desirable to achieve good signal-to-noise ratios. Particularly interesting is the redox reactivity of certain *N*-oxides in biological systems. It has stimulated the development of hypoxia-activated prodrugs for cancer therapy in the past and has recently been transferred to antibacterial treatments. The selective activation of *N*-oxides by reductive metabolism in hypoxic environment can also be used for targeting purposes, and the combination with other therapeutic concepts are promising. A number of very recent examples have shown that additional hypoxia targeting improves the efficacy and the therapeutic window for cytotoxic drugs or other therapeutic concepts such as starvation therapy or photodynamic therapy. These new combined approaches might also be the key to translate *N*-oxide-based hypoxia-activated drugs into the drug market, which has not been successful so far. An extremely promising field of application is the use of polymeric *N*-oxides as hydrophilic polymers for drug conjugation and materials for biomedical applications. Although polymeric *N*-oxides have been principally known for quite a while, their application as stealth materials, for targeting purposes or substitutes of PEG for drug conjugation, is a very recent development. It has stimulated a number of high-quality studies through the last years. It is notable that many applications of polymeric *N*-oxides rely on their stealth characteristics and require chemical and enzymatic inertness of the polymers. However, *N*-oxides, which have intrinsic redox reactivity, are also sensitive to higher temperatures. Whether these properties translate into cytotoxicity or material incompatibilities for example with sterilization protocols remains to be evaluated in many cases. This intrinsic reactivity can also be advantageous for targeting or combined stealth and antimicrobial approaches. A lot of interesting applications of polymeric *N*-oxides can therefore be expected in the biomedical field.

## ABBREVIATIONS USED

AMMOFs: metal–organic framework nanosystem combining AQ4N and Mn(II)
CPT: Camptothecin
DTPA: diethylene-pentaacetate
EDC: 1-ethyl-3-(3-(dimethylamino)propyl)carbodiimide
FMO: flavin-containing monooxygenase
FRP: free radical polymerization
GOx: glucose oxidase
HAP: hypoxia-activated prodrugs
HDL: high-density lipoprotein
HIFU: high-intensity focused ultrasound
IFA: interferon alpha
mCPBA: *meta*-chloroperoxybenzoic acid
MRC: mitochondrial respiratory chain
Mtb: *Mycobacterium tuberculosis*
NPs: nanoparticles
PC: propargylcarbamate
PDHK1: pyruvate dehydrogenase kinase 1
PDT: photodynamic therapy
PDMA: poly(2-(*N,N*′-dimethylamino)-2-ethyl methacrylate)
PODMA: poly(2-(*N*-oxide-*N,N*′-dimethylamino)-2-ethyl methacrylate)
PTX: paclitaxel
SI-ATRP: surface-initiated atom transfer radical polymerization
TMAO: trimethylamine-*N*-oxide
TPZ: tirapazamine
